# School-based mental health promotion: A global policy review

**DOI:** 10.3389/fpsyt.2023.1126767

**Published:** 2023-04-17

**Authors:** Margaretha Margaretha, Peter Sebastian Azzopardi, Jane Fisher, Susan Margaret Sawyer

**Affiliations:** ^1^Department of Paediatrics, Melbourne Medical School, Faculty of Medicine, Dentistry and Health Sciences, University of Melbourne, Parkville, VIC, Australia; ^2^Centre for Adolescent Health Royal Children’s Hospital, Melbourne, VIC, Australia; ^3^Murdoch Childrens Research Institute, Melbourne, VIC, Australia; ^4^Faculty of Psychology, Universitas Airlangga, Surabaya, East Java, Indonesia; ^5^Adolescent Health and Wellbeing, Telethon Kids Institute, Adelaide, Australia; ^6^School of Public Health and Preventive Medicine, Faculty of Medicine, Nursing & Health Sciences, Monash University, Melbourne, VIC, Australia

**Keywords:** school, mental health promotion, United Nations (UN), guideline, manual, global policy

## Abstract

**Objectives:**

Schools are increasingly recognized as important settings for mental health promotion, but it is unclear what actions schools should prioritize to promote student mental health and wellbeing. We undertook a policy review of global school-based mental health promotion policy documents from United Nations (UN) agencies to understand the frameworks they use and the actions they recommend for schools.

**Methods:**

We searched for guidelines and manuals from UN agencies through the World Health Organization (WHO) library, the National Library of Australia and Google Scholar, from 2000 to 2021, using various combinations of search terms (e.g., mental health, wellbeing, psychosocial, health, school, framework, manual, and guidelines). Textual data synthesis was undertaken.

**Results:**

Sixteen documents met inclusion criteria. UN policy documents commonly recommended a comprehensive school-health framework aimed at integrating actions to prevent, promote, and support mental health problems within the school community. The primary role of schools was framed around building enabling contexts for mental health and wellbeing. Terminology was relatively inconsistent across different guidelines and manuals, particularly around how comprehensive school health was conceptualized, which included aspects of scope, focus, and approach.

**Conclusion:**

United Nations policy documents are oriented toward comprehensive school-health frameworks for student mental health and wellbeing that include mental health within wider health-promoting approaches. There are expectations that schools have the capabilities to deliver actions to prevent, promote and support mental health problems.

**Implication:**

Effective implementation of school-based mental health promotion requires investments that facilitate specific actions from governments, schools, families, and communities.

## Background

Mental health and wellbeing are recognized as major contemporary public health challenges, including in school-age children and adolescents (1–4). Students with mental health disorders are also appreciated to have poorer academic attainment, which suggests that addressing mental health and wellbeing is important for both health and education outcomes (5–7). In this context, schools are expected to act as settings that can deliver actions to promote positive mental health and wellbeing, prevent mental disorders, as well as manage student mental health needs, including identification, referral, and provision of support (8–11).

Guidelines and manuals can assist schools to develop their capacity to identify, prioritize, and deliver evidence-based, feasible, and contextualized approaches to mental health promotion for children and adolescents. Over the past few decades, various policies, guidelines, and manuals have been developed by governmental and non-governmental organizations at national, regional, and international levels. Globally, the United Nations (UN) agencies have maintained focus on promoting adolescent mental health and wellbeing among its member states by documenting policies in the form of guidelines and manuals. Several UN agency guidelines address mental health within broader approaches to school-health services or health promotion in schools, such as the World Health Organization (WHO) Global School Health Initiative, which first developed guidelines for Health-Promoting Schools in 1995 (12–14) and more recently developed global standards and indicators for Health-promoting Schools and an accompanying implementation guidance (15, 16). Other policies specifically focus on mental health and include the value of orienting member states and national governing bodies toward more preventive and promotive approaches to mental health and wellbeing in schools. This includes efforts by the United Nations Educational Scientific and Cultural Organization (UNESCO) and United Nations Children’s Fund (UNICEF) that require schools to assist students to develop appropriate social and emotional skills and engage in positive classroom behaviors (17, 18).

These UN policy documents are intended to serve as references for implementation but risk being inhibited by several challenges. First, each UN agency’s documents potentially speak to different audiences and sectors. UNICEF, for example, primarily speaks to social protection audiences, while UNESCO targets the education sector, and WHO is oriented to the health sector. Further uncertainty is around the extent to which these documents use common frameworks or approaches toward school-based mental health promotion. An additional challenge is that the language of “social and emotional wellbeing” is more typically used by the education sector than “mental health,” which is more commonly used by the health sector. These factors may lead to difficulties aligning different documents and inadvertently contribute to unclear expectations of schools’ roles in promoting mental health and wellbeing.

Given the potential breadth of this policy landscape and the implications of this for the multiple actions that schools could potentially take to promote mental health and wellbeing, we set out to identify what mental health promotion frameworks are used within the various global guidelines and manuals of school-based mental health promotion with the objective of understanding the specific actions schools can take to promote student mental health and wellbeing. Specifically, this study aimed to address two research questions: (1) To what extent are common frameworks used across the UN agency guidelines and manuals that are relevant for school-based mental health promotion? and (2) How should schools facilitate mental health promotion? We undertook a policy review that aimed to synthesize the key recommendations within global manuals and guidelines to develop an integrated understanding of the potential role of schools in promoting the mental health and wellbeing of school-age students.

## Methods

We set out to review all UN agency manuals and guidelines of relevance to school-based mental health promotion. In this context, we defined a guideline as a report that consists of general principles that provide assistance in making decisions for specific circumstances or that give direction in setting standards or determining a course of action (19, 20). We defined a manual as a collection of instructions on how to perform an activity, which generally serves as a more practical resource or reference (21). The production of school mental health guidelines and manuals is a public health policy approach used by UN agencies; such documents are widely acknowledged for their credibility, practicality, and global relevance, both for high-income countries (HIC) and low-middle-income countries (LMIC). For this reason, this study focused on identifying UN agencies’ manuals and guidelines that were intended to be globally relevant.

### Search strategy

Given our interest in scoping globally relevant manuals and guidelines intended to support mental health promotion in schools (22), we searched for reports from Google Scholar, through the National Library of Australia^1^ and using the Institutional Repository for Informational Sharing–World Health Organization (WHO-IRIS) library for documents published by the relevant UN agencies over the last two decades. We searched through WHO-IRIS as most health-related policy documents are stored in this platform, regardless of the UN agency that produces them. We aimed to identify reports published from 2000 to 2021, inclusive. One reviewer conducted the search between 1 May 2021 and 16 May 2022. The search strategy used a combination of the following terms: “mental health,” “wellbeing,” “psychosocial,” “health,” “mental disorder,” “mental illness,” “school,” “education,” “school health,” “children,” adolescent,” “manual,” “guidelines,” “policy,” and “framework” (see Supplementary Box S1 for the search strategy).

### Eligibility criteria

We defined eligible documents as any UN agency guideline and manual on school-based mental health promotion published in English from 2000 to 2021. We excluded technical reports, country-level reports, regional-level reports, meeting reports, statistical reports, epidemiological reports, media resources, research papers, reviews of studies, editorials, books or codex, non-English texts, academic theses, and other texts.

### Textual data analysis

The Joanna Briggs Institute’s guidance for synthesizing evidence from narrative, text, and opinion-based evidence was used for data synthesis for this policy review. Specifically, data synthesis was undertaken using a three-step categorization strategy, as detailed in McArthur (23). The first step is the conclusion process, where a conclusion for each document is generated to answer a research question. The second step is the categorization process, in which the reviewers read all conclusions and identify similarities that could be used to create one or more categories encompassing the various findings from the first step. The third step involves synthesizing findings, where the reviewers undertake a meta-synthesis of all categorized findings. The synthesizing is based on their expert opinion for generating a set of comprehensive statements that resemble important information in the review. The textual analytic process was generated into the NOTARI view for tabulation, which sorts conclusions and categorizations within their unitary synthesized finding to adequately represent the data (23).

We used the multi-tiered model of school-based mental health intervention as the framework for data extraction (9). The reasons for this are that: it has a long history of use; is relevant for both the education and health sector workforces across primary and secondary schools; and has the broadest scope (it addresses mental health needs that range from health promotion of relevance to the entire student body, to more targeted approaches for those at risk, as well as specific responses for the minority with a clinical diagnosis of mental disorder) (9, 24).

## Results

**Figure 1:**
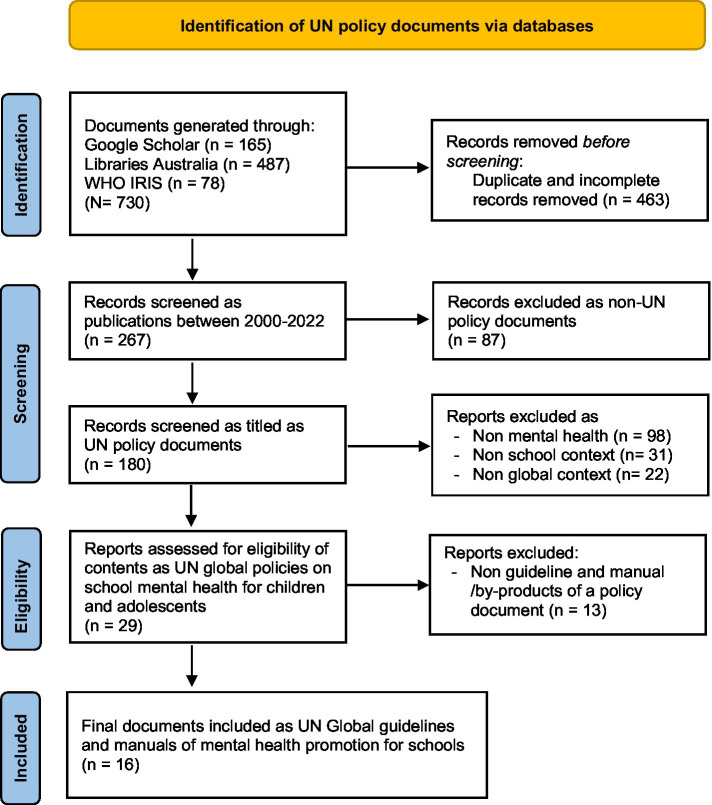
Schematic representation of the search process.

The search identified 730 records, from which 16 globally relevant manuals and guidelines were included in the final policy review. Figure 1 outlines the search process referring to the PRISMA flow diagram (25), and the selected documents are listed in Supplementary Box S2. The largest number of reports (*n* = 12) came from WHO, with others from UNESCO, UNICEF, and collaborations of several UN agencies and their partners. A summary of relevant findings from these documents are listed in Table 1. These 16 globally oriented mental health policy documents span a wide scope of school-based mental health promotion, ranging from those that focus on universal or population approaches to health promotion to more selective and indicated interventions.

**Table 1:** Summary of relevant findings from the included UN agencies’ policy documents on school-based mental health promotion.

No	Document*	Author(s)	Goal	Recommendations	Relevant information
1	WHO (2000) Local Action for creating Health-Promoting Schools (HPS) (Manual)	A partnership of WHO (Department of Noncommunicable Diseases Prevention and Health Promotion; NCD-DVIP), UNESCO and Education Development Centre Inc. (EDC).	To assist schools and community leaders in identifying health issues faced by the school and the community and making the appropriate steps for solving health issues through schools to improve student health and learning.	WHO recommends its member states support local action in creating Health-Promoting Schools (HPS): 1. Getting started: building local support 2. Taking actions: a. Establishing core teams: School health team and Community advisory committee b. Assessing community health problems, policies and resources c. Setting goals, objectives and action plan d. Collecting information and demonstrating progress e. Linking local efforts to larger initiatives.	This manual is one of the WHO’s Information Series on School Health. Six implementation strategies for HPS were: 1. Engage health, education, and community leaders 2. Provide a safe, healthy environment (physical and psychosocial) 3. Provide skills-based health education 4. Provide access to health services 5. Implement health-promoting policies and practices 6. Improve the health of the community
2	WHO (2000) Preventing Suicide: A resource for teachers and school staffs. (Manual)	A partnership of WHO (Department of Mental Health and Substance Abuse; MHSA) and its international collaborators.	To equip teachers and school staff to implement suicide prevention strategies in school settings.	WHO recommends schools implement suicide prevention strategies integrated within an HPS framework. 1. General prevention 2. Strengthening the mental health of teachers and staff 3. Strengthening students’ self-esteem 4. Promoting emotional expression 5. Preventing bullying and violence at school 6. Providing information about care services 7. Intervention when suicide risk is identified 8. Developing trustful communication 9. Referral to professionals 10. Removal of potential means of suicide from distressed students.	This manual is one of the WHO’s Suicide Prevention resource series (SUPRE). WHO updated the suicide prevention framework with the National Suicide Plan for Countries and Live life in 2021.
3	UNESCO (2002) Focusing Resources on Effective School Health (FRESH): A comprehensive school-health approach to achieve Education for All (Guideline)	A partnership of experts from UNESCO, UNICEF, WHO, the World Bank and Education International	To assist national governments to improve student’s learning outcomes by incorporating school-health approaches through FRESH in the National Education plans.	UNESCO recommends its member states implement FRESH’s four core components in all schools: 1. School-health policies. 2. Healthy learning environment: water, sanitation and safety. 3. Skills-based health education 4. Health and nutrition services In addition, there three FRESH supporting activities: 1. Effective partnerships between teachers and health workers (education and health sectors) 2. Effective community partnerships 3. Pupil awareness and participation	This guideline was followed by a guideline in 2013: Monitoring and Evaluation guidelines: Eight indicators to support FRESH implementation.
4	WHO (2003) Creating an Environment for Emotional and Social Wellbeing (Manual)	A partnership of WHO (NCD-DVIP, MHSA and Evidence and Research), UNICEF, and EDC.	To introduce the Psychosocial Environment (PSE) self-assessment tool and assist schools in creating a healthy psychosocial environment that can enhance students’ learning and social and emotional wellbeing.	WHO recommends teachers and school leaders assess the situation in their school by using the PSE profile and to make any organizational changes that would assist in promoting a healthy psychosocial school environment within the HPS approach.	This manual is one of the WHO’s Information Series on School Health.
5	WHO (2003) Family life, Reproductive health, and Population education (FRPE) (Manual)	A partnership of WHO (NCD-DVIP), UNICEF, and EDC.	To assist schools in creating school-based efforts to educate young people about family, reproductive health, and population issues to prevent related health problems, such as unintended and early pregnancies, HIV/STI, and sexual violence.	WHO recommends its member states implement FRPE through the HPS approach. 1. Establish core teams (school-health team and community advisor committee) 2. Gain support and commitment from various stakeholders (government and policies, family and community, school members, and youth participation) 3. Conduct situational analysis (need and resources assessment) 4. Action planning (goals, objectives, activities, and monitoring-evaluation)	This manual is one of the WHO’s Information Series on School Health.
6	WHO (2003) Skills for Health (Manual)	A partnership of WHO (NCD-DVIP), UNICEF, and Health and Human Development Programs at EDC.	To guide governments, education and health workers to improve youth health education through skills-based health education, including life skills.	WHO and UNICEF recommend their member states to implement health education through skills-based health education through HPS, FRESH, and Child-Friendly School strategies.	This manual is one of the WHO’s Information Series on School Health and UNICEF’s FRESH resources.
7	UNICEF (2009) Child-Friendly School (CFS) Manual (Manual)	UNICEF Education Section staff and experts from partner agencies.	To assist governments and educators for improving the quality of education systems by introducing and guiding the implementation of CFS.	UNICEF recommends its member states adopt the CFS concept and implement it in their contexts. CFS is a model that aims to provide healthy, safe, and protective schools staffed with trained teachers and equipped with appropriate resources and conditions for optimal learning.	CFS aimed to be a theoretical model and practical reference of the intersectoral intervention approach in education.
8	UNESCO (2016) Education for health and wellbeing: Contributing to the Sustainable Development Goals (Guideline)	UNESCO Section for Health and Education led an extensive consultation process with its partners.	To assist governments to include the comprehensive sexuality-HIV education and health and youth wellbeing promotion in their National Education plans.	UNESCO recommends its member states ensure that young people have access to two strategic priorities: 1. A good quality, comprehensive sexuality and HIV education. 2. A safe, inclusive, health-promoting learning environment.	Partnerships with a range of actors and other UN agencies are considered central for UNESCO’s implementation strategy.
9	WHO (2016) INSPIRE Handbook: Seven strategies for ending violence against children (Guideline)	A partnership of WHO (NCD-DVIP) and UNICEF, with the United States Centers for Disease Control and Prevention (CDC), the Pan American Health Organization (PAHO), End Violence Against Children, the President’s Emergency Program for AIDS Relief (PEPFAR), United Nations Office on Drugs and Crime (UNODC), United States Agency for International Development (USAID), and World Bank.	To assist governments and communities in preventing and responding to violence against children and adolescents in their respective countries.	WHO and UNICEF recommend their member states implement the seven evidence-based strategies and monitor changes over time to prevent and reduce violence against children. 1. Implementation and enforcement of laws 2. Norms and values 3. Safe environments 4. Parent and caregiver support 5. Income and economic strengthening 6. Response and support service 7. Education and life skills.	This guideline is complemented by the INSPIRE Indicator guidance and results framework (2018). INSPIRE complements the Global Plan of Action to strengthen the role of the health system within a national multisectoral response to address interpersonal violence against women and children in 2016.
10	WHO (2016) Mental Health Global Action Programme-Intervention Guide (mhGAP): Intervention Guide-version 2.0: (Manual)	WHO (MHSA) and its international partners.	To assist national and local non-specialized health care providers in providing a set of good clinical practices for people seeking health support for Mental, Neurological and Substance use disorders (MNS) in non-specialized health settings.	WHO recommends its member states implement assessment, intervention and prevention of MNS in non-specialized settings within their National or Local Health Plan. Health systems and providers can use the mhGAP operational manual as practical, step-by-step guidance for integrating mental and physical health services.	An example of a comprehensive policy consisting of a guideline and a manual. This manual is an updated document from the mhGAP guideline in 2008 and an intervention guide-version 1.0 in 2010.
11	UNICEF (2018) Operational guideline: Community-Based Mental Health and Psychosocial Support in Humanitarian Settings (CB-MHPSS) (Guideline)	UNICEF, with guidance from the Inter-Agency Standing Committee in Mental Health and Psychosocial Support in Emergency Settings (IASC-MHPSS).	To guide UNICEF staff, partners, and humanitarian agencies to help effectively and implement CB-MHPSS in order to promote a safe and nurturing environment for children’s recovery, wellbeing and protection.	UNICEF recommends agencies in humanitarian settings to implement layered mental health and psychosocial support when working with children. 1. Basic service and security to ensure the dignity and wellbeing for all children and community. 2. Family and community support for recovery and strengthening resilience. 3. Focused care with non-specialized supports. 4. Specialized care by clinicians.	UNICEF’s CB-MHPSS adopted the multi-tiered intervention model from IASC-MHPSS in 2007 which is equivalent with WHO multi-tiered model 1994.
12	WHO (2019) Accelerated Action for the Health of Adolescents (AA-HA!) (Guideline)	A partnership of WHO, UNICEF, UNESCO, UNFPA, UN-WOMEN, USAID, and World Bank.	To guide member states and the related stakeholders in planning, organizing and facilitating the development of national comprehensive multisectoral adolescent health strategies and plans aligned with their national health plan.	WHO recommends its member states develop their own national adolescent health strategies and plans. 1. Planning the process for developing a national adolescent health strategy and plan 2. Preparing a background document on adolescent health and policies 3. Technical working group workshop 4. First national AA-HA! workshop 5. Finalization of the national adolescent health strategy, implementation plan, monitoring and evaluation. 6. Costing the implementation plan.	This guideline is designed for governments, policymakers and groups of stakeholders at the national level.
13	WHO (2019) School-based violence prevention: A practical handbook (Manual)	WHO (NCD-DVIP), UNICEF, and UNESCO.	To assist schools and education systems in providing education and organized activities for children to prevent violence through a whole-school approach.	WHO recommends schools to be able to perform violence prevention: 1. Develop leadership, policies and coordination methods 2. Collect data on violence and monitoring strategies 3. Prevent violence through curriculum-based activities 4. Work with teachers on values and beliefs and train them in positive discipline and classroom management 5. Respond to violence when it happens 6. Review and adapt school buildings and grounds 7. Involve parents in violence prevention activities 8. Involve the community in violence prevention 9. Evaluate violence prevention activities and use the evidence to strengthen your approach.	This manual operationalizes the guidance for schools in dealing with violence against children and interpersonal violence against women and children. This manual is an updated module of WHO’s Information Series on violence prevention in school from 1998.
14	WHO (2020) Helping Adolescents Thrive (HAT): Guidelines on mental health promotive and preventive interventions for adolescents (Guideline)	A partnership of WHO and its UN partners.	To provide evidence-informed recommendations for governments, education and health providers for facilitating mental health promotion and mental disorders prevention for adolescents aged 10–19 years in schools and communities, and digital platforms.	WHO recommends its member states implement evidence-based mental health interventions in a stepped-care model: 1. Universal interventions for all adolescents; 2. Targeted interventions for adolescents at increased risk of mental disorders; 3. Indicated interventions for adolescents who present early signs and/or symptoms of mental disorders.	HAT complements AA-HA! and mhGAP guidelines on adolescent health, particularly for facilitating promotive and preventive mental health interventions.
15	WHO (2021) Making every school a Health-Promoting School: Implementation Guidance (Guideline)	A partnership of WHO and UNESCO.	To assist national, sub-national, and local governments in developing, planning, funding, and monitoring sustained whole-school approaches.	WHO and UNESCO recommend their member states support HPS implementation through attending to eight global standards: 1. Government policies and resources 2. School policies and resources 3. School governance and leadership 4. School and community partnerships 5. School curriculum 6. School social–emotional environment 7. School physical environment 8. School-health services This guidance promote using an implementation cycle for continuous improvement.	This guidance accompanies the report on global standards and indicators for HPS (2021), a report that builds on earlier documentation. The target audience is at a government level, with the intention of supporting member states to develop national HPS plans.
16	WHO (2021) WHO Guideline for School Health Services (SHS) (Guideline)	A partnership of WHO and UNESCO.	To support national governments and international partners to develop effective, evidence-informed SHS programs to better meet the health and development needs of school-age children and adolescents.	WHO and UNESCO recommend that member states improve their national school-health policies and programs by referring to the listed evidence-based intervention that can be delivered by comprehensive school-health services. SHS can operate through implementation of two complementary approaches: (1) School-based health services (within the school); and (2) school-health linked services (beyond the school).	This guideline encourages local-level and school implementations through seven activities: 1. Health promotion 2. Health education 3. Screening leading to care/referral and appropriate support 4. Preventive intervention 5. Clinical assessment leading to care/referral and appropriate support 6. Health services management 7. Support for other pillars of HPS

^*^UN global policy documents are chronologically ordered.

Seven different global school mental health policy approaches were identified: (1) Health-promoting Schools, a Global School Health Initiative from WHO that has recently been widely endorsed by other UN agencies, most notably UNESCO (13, 15); (2) the Child-Friendly Schools initiative from UNICEF (18); (3) the inter-agency initiative of Focusing Resources for Effective School Health (FRESH), from which the renewed focus on Health-promoting Schools has also emerged (17); (4) the INSPIRE Seven strategies for ending violence against children (26); (5) the Mental Health Gap Action Programme (27); (6) Accelerated Action for the Health of Adolescents from WHO (28); and (7) Helping Adolescents Thrive, a UN multiagency initiative (29). Three of these seven policy approaches were designed for schools and explicitly promoted a comprehensive school-health framework that acknowledges the importance of addressing mental health within a wider scope of health topics (13, 17, 18). The remaining four also acknowledge the importance of schools in attending to diverse health needs.

As might be expected, several of these policy documents show evidence of progression over time. For example, the WHO’s Health-promoting Schools framework shows progress from its early conceptualization in 2000 (13) into a set of eight global standards and indicators (15). Likewise, UNESCO’s FRESH progressed from an earlier guideline in 2002 (17) that advanced assessment strategies and tools by including detailed monitoring and evaluation guidelines in 2013 (30). There is also increasing evidence of inter-agency engagement. For example, the approach of Health-promoting Schools was first developed by WHO but has been redeveloped and co-branded with UNESCO (13, 16). Similarly, mhGAP-IG 2.0 (31) is the latest guideline and manual from WHO for reducing mental, neurological, and substance use disorders that follows earlier guidelines on the Mental Health Gap Action Program from 2008 and 2010 (32, 33).

### Commonalities within the global manuals and guidelines

**Figure 2:**
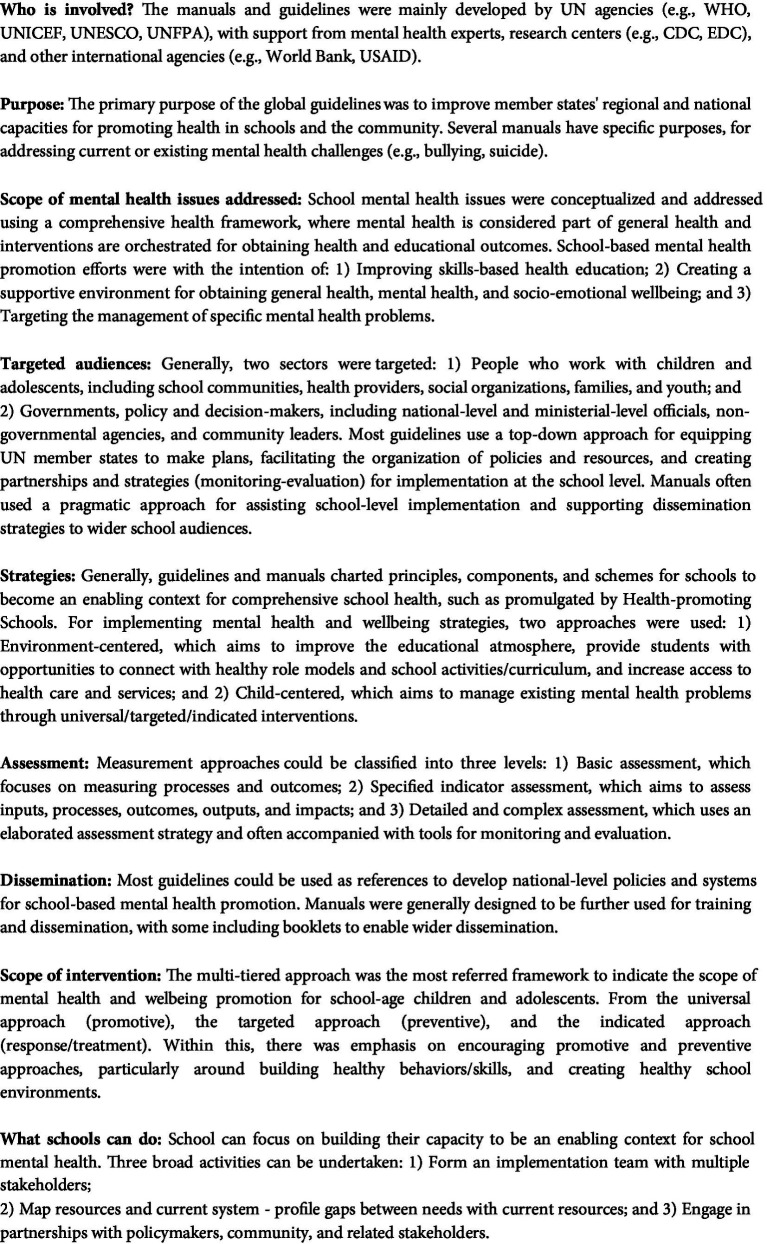
Summary of key points of global UN agencies’ mental health guidelines and manuals.

In general, we found much in common across these UN global policy documents (see Figure 2 for a summary of key points). UN policies on school-based mental health were predominantly oriented around a comprehensive school-health framework that is intended to holistically improve the health and wellbeing of all school community members, including students and teachers, as well as the wider community. Another common element within this framework is the recognition that collaboration between schools, governments, families, communities, and related stakeholders is required to implement comprehensive approaches to mental health promotion in schools.

Several manuals were developed to address more specific challenges facing schools and their communities, such as youth suicide (34), violence against children (26, 35, 36), and crisis interventions (37). We found that both generic mental health promotion documents and these more specific documents promote a comprehensive framework and acknowledge the diversity of mental health needs within any school community (13, 15–18, 38, 39). There are also clear expectations that schools are able to attend to a wide array of mental health needs within the school community. Schools are also expected to have the capacity to intervene when required to manage more specific mental health concerns, either for individuals (e.g., depression) or around common issues for a school community (e.g., climate crisis anxiety) (37, 40). The importance of a school’s social environment for mental health was also recognized (18, 41, 42). Schools are encouraged to facilitate preventive and promotive approaches through developing healthy social environments and providing activities to improve students’ mental health literacy and communication skills (43).

Another common feature was that these guidelines and manuals explicitly require the involvement of multiple stakeholders including school administrators, government organizations, community, and non-government organizations (NGOs). This requires action by schools to develop or improve functional networks with relevant related stakeholders, particularly governments and communities.

United Nations guidelines are intended to assist national and local governments to make plans, facilitate policies and supplies, and generate coordination and monitoring strategies. Consistent with this, several guidelines used a “top-down approach,” which was particularly evident around UN member states’ commitment to national school-health plans (15, 44, 45). In comparison, manuals are designed for more practical purposes and targeted schools themselves, with the goal of providing schools with tools that might assist them to implement mental health promotion programs (34, 42).

The manuals and guidelines clearly outlined principles, mechanisms, and systems for schools to promote health comprehensively and identified inclusive strategies to address mental health and wellbeing. Part of the comprehensiveness of the framework is that strategies for promoting mental health and wellbeing spanned from preventively oriented approaches to health promotion while also acknowledged the importance of access to health services. There were two elements to this comprehensive approach. The first was environment-centered, focused on improving the educational and relational atmosphere or ethos within a school (18, 42). The second was student-centered, focused on individual and problem-focused approaches, such as resilience-building or interventions to improve individual coping skills, social support, and self-esteem (39, 43). This latter approach also included access to health services when required. These two approaches were viewed as complementary to each other.

Most manuals suggested that structured approaches were needed to support dissemination, such as assessment tools and the inclusion of practical material for training. For example, several manuals included training modules to help deliver transferrable knowledge (concepts, principles, and strategies about program implementation) and equip learners with the skills and tools to apply this (13, 31, 35, 42). Most guidelines were appreciated as valuable references to relevant concepts, principles, and strategies. Within these, more typical approaches to dissemination, such as promoting the transferability of skill sets, were generally not described. Guidelines seemed particularly useful in informing policymakers and UN member state governments around developing national policies (17, 36, 38).

These globally oriented guidelines and manuals require national commitments for recommendations to be implemented, which is also supported by recommendations around monitoring and evaluation. These documents consistently included some recommendations for monitoring and evaluation, although these varied in their approaches. Some recommended a somewhat basic assessment consisting primarily of process and outcome evaluations (13, 41), while others recommended a broader assessment of program logic indicators, such as input, process, outcome, output, and impact evaluation (30, 37, 46). Several manuals provided a list of measurement tools and gave examples for developing an assessment approach at the school level, but without being framed within a wider approach to monitoring and evaluating national dissemination (39, 42, 43). A few recommended more complex assessment approaches and included strategies and assessment tools within the documentation (15, 46). The most comprehensive assessment strategy was articulated within the UNESCO FRESH model, which outlined different levels of assessments that ranged from the national level for assessing the existence, and the quality of national health education plans to the school level for assessing the extent that schools implemented skills-based education (30). These documents clearly expect schools to have the capacity to provide mental health promotion according to the level of mental health needs of the school community. This suggests that national and local assessments (e.g., school and community surveys) that enable school-based actions to match local needs are required, including knowledge of risk factors for mental health problems, as articulated in the new Global Standards and Indicators for Health-promoting Schools and Systems (15).

Over the two decades of this review, the clearest commitment around the scope of mental health prevention and promotion was to deliver universally framed interventions that aimed to reach all members of the school community (rather than targeting interventions for more at-risk students or to those identified with mental health problems). The universal approach appears to have been widely interpreted as reflecting aspects of school curricula (e.g., individual student-focused approaches to developing skills-based health education) and extra-curricular activities for students, together with efforts to promote the establishment of healthy school environments. Less frequently noted across policy documents was the need to promote the mental health and wellbeing of all school community members, including teachers and school staff, which was particularly noted in the new Global Standards and Indicators for Health-promoting Schools and Systems (15, 16).

### What schools can do to promote mental health

These UN guidelines and manuals suggest that the role of schools is mainly focused on building enabling contexts for mental health and wellbeing. Within these documents, three strategies were recommended for schools to create the enabling structures, functions, and roles that underpin a supportive school system (47, 48). These were to:

1. Establish a school mental health implementation team. Schools can form a school mental health team, as a part of the school-health system, which may include school leaders, teachers, school-based mental health staff, and special education teachers. The school mental health team also needs to develop a network of connections within a school’s local community, which might include local health services, families, civil society organizations, and youth groups;
2. Profile existing needs, resources, and systems. Schools can conduct a mapping exercise as a strategy to identify gaps between needs and current resources, including accessibility. This will be relevant for approaches that aim: to identify students who may benefit from health services, to identify relevant curriculum materials, such as those that may help build mental health literacy and reduce stigma around people with mental health conditions, and to consider the role of the school’s social and physical environments in promoting wellbeing;
3. Partner with policymakers and the community. Schools are required to maintain cooperative engagement with governments and related stakeholders, as beyond ensuring that standards and regulations are implemented, access to resources and personnel are also needed.

### Multiple meanings of “comprehensive school health”

**Figure 3:**
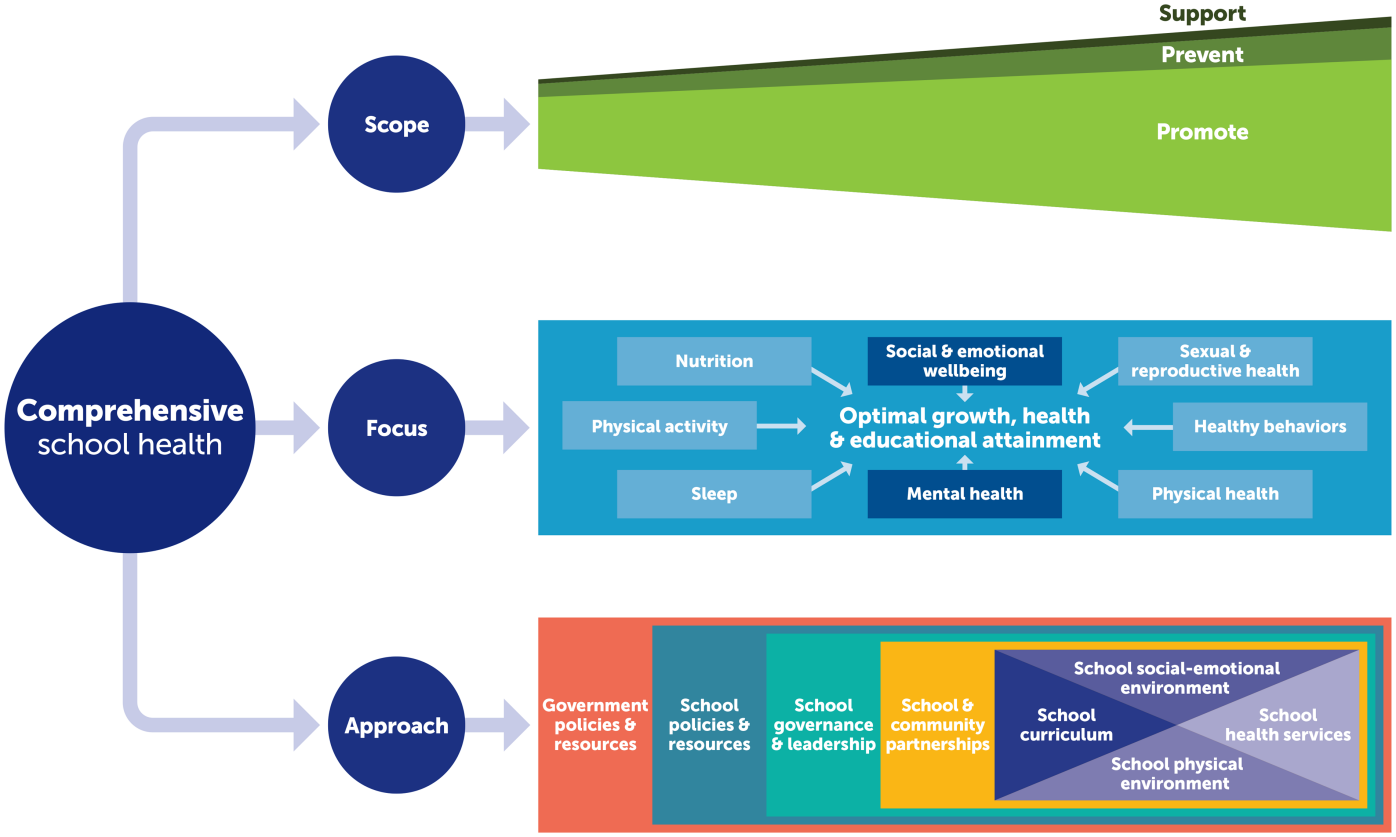
Elements of comprehensive school health included aspects of scope, focus, and approach.

While a comprehensive school-health framework was commonly articulated within these documents, we identified three different conceptions of “comprehensive,” as shown diagrammatically in Figure 3. The first derives from the 1994 multi-tiered mental health model, which conceptualized a comprehensive suite of interventions that spanned from universally oriented health promotion and preventive interventions to more indicated interventions that support students with mental health issues at school (9). In this way, the notion of comprehensiveness is around scope, with the multi-tiered mental health model oriented to and inclusive of different populations, embodying different needs and risks.

A second concept, embodied within WHO’s more recent Health-promoting Schools policy documents, views comprehensiveness in relation to the focus of health addressed by school health. In this way, comprehensive health promotion conceptualizes mental health promotion as one of a range of health topics that schools need to address. For example, the Health-promoting Schools approach encourages schools to respond to the health priorities within its community, which beyond mental health might include aspects of nutrition, physical activity, unintentional injury and interpersonal violence, substance use, self-harm, gender norms and sexual and reproductive health, communicable and non-communicable disease, physical and sensory disabilities, and oral health (9, 15). A feature of this approach is its attention to the efficiencies that can be gained by taking a more comprehensive view of health; strategies to promote physical activity can be framed as mental health promotion, as can efforts to address more healthy gender norms, responses to student and teacher bullying and interpersonal violence at school. This approach also appreciates that attending to more generic aspects such as student–student relationships (e.g., zero bullying), student–teacher interactions (e.g., approaches to punishment and student recognition), and active pedagogy (e.g., leadership opportunities) can enhance school connectedness which benefits mental health.

The third conceptualization of comprehensive, embodied within FRESH and also within Health-promoting Schools, views comprehensive school health as an approach or framework for addressing health, including mental health, in a planned, integrated, and holistic approach while aiming to improve students’ educational outcomes (17). Rather than a focus on a particular population, or health topic, this notion of comprehensive refers to the breadth of approach, which is whole-school in its orientation and explicitly intended to promote health and wellbeing as well as educational attainment.

A further finding was the inconsistency in orientation and terminology between these guidelines and manuals, even within the same agency. For example, within WHO, one manual for mhGAP was referred to as an “intervention guide” (31) while another manual was called a “practical handbook” for school-based violence (35). UNICEF refers to its Child-Friendly School’s policy document as a manual, yet while its contents indeed focus on implementation (as expected from a manual), the inclusion of principles, indicators, and standards, and country case studies appear more appropriate content for a guideline (18).

## Discussion

**Figure 4:**
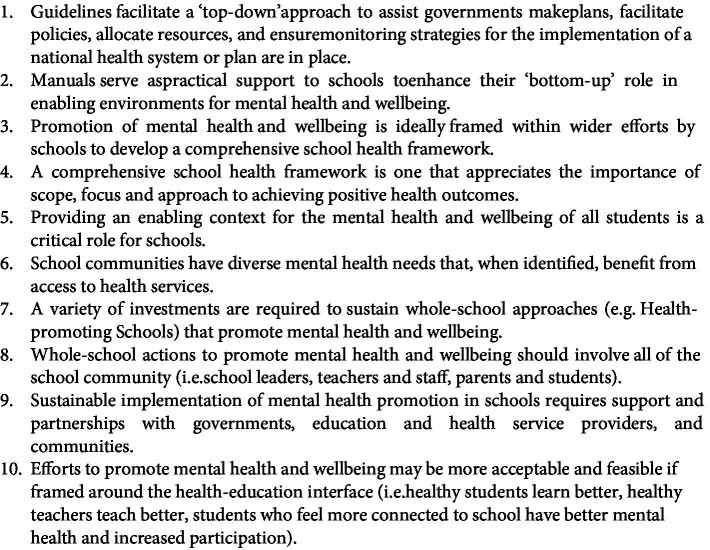
Key recommendations synthesized from the 16 UN agencies’ guidelines and manuals.

This policy review of global manuals and guidelines reveals that the UN agencies have made significant efforts to orient national governments and their schools to the importance of promoting the mental health and wellbeing of school-age children worldwide, a feature that has gained acute significance since the COVID-19 pandemic. We identified three major findings from this synthesis of 16 UN agency global policy documents on school mental health promotion. The first finding was that efforts to promote mental health are ideally framed within wider efforts by schools around health promotion. The second finding was the importance of schools engaging in health-promoting actions that are universal in their scope, that is, are aimed at all students. Linked to this is the third finding, which is the important role that schools have in providing an enabling context for mental health and wellbeing. A series of recommendations arising from this policy review are described in Figure 4.

These findings suggest the value of embedding a school mental health strategy within a school’s comprehensive health promotion framework, which will ideally appreciate the importance of scope, focus, and approach to achieving positive health outcomes. Such a framework reinforces that schools need to be able to deliver actions to address mental health by implementing strategies to prevent mental health problems, promote wellbeing, and manage students with mental disorders, with an eye to how these strategies intersect with other health issues and themes (e.g., gender norms, safety, nutrition, physical activity). Attending to student mental health exemplifies the complex relationships between health and educational attainment, given the effects of mental disorders on student motivation and attention, school engagement, absenteeism, early school completion and learning outcomes, and the knowledge of how school connectedness predicts mental health (15, 16, 49). Investment will be required to orient school communities to this knowledge, as without this, an obvious challenge for schools is that addressing health concerns may not be perceived as a priority by those in the education sector, including families and communities (50).

Sustainable implementation of mental health promotion in schools will require investment by governments in school communities to equip them with the resources they need to actively engage in whole-school approaches (50, 51). One challenge is that many of the guidelines and manuals we reviewed used more individually-oriented approaches (e.g., curriculum-based interventions to promote socio-emotional learning). Using all the levers available to schools to promote mental health is required, including approaches that engage a school’s social mechanisms to promote connectedness and belonging (49).

Collaboration between the UN agencies creates opportunities for more specific guidelines around mental health. In this way, for example, WHO’s mental health Gap Action Program-Intervention Guide (mhGAP-IG) can be seen to intersect with more generic approaches such as Accelerated Action for the Health of Adolescents (AA-HA!), INSPIRE, and Health-promoting Schools. Similarly, UNICEF has promulgated efforts to address mental health in specific contexts, such as humanitarian settings, through the Inter-Agency Standing Committee Guidelines in Mental Health and Psychosocial Supports (IASC-MHPS) (52, 53), which has developed a module on Community-based Mental Health and Psychosocial Support in Humanitarian Settings (CBMHPSHS). While these approaches were not explicitly created for school contexts, they are also relevant for promoting mental health in schools. Given these opportunities, it would be pleasing to see future generic policy documents articulate how they link to specific guidelines, and vice versa, as articulated by at least some of these documents (37).

This policy review suggests that over the past two decades, there has been a growing trend toward advocating universal approaches that target all school populations (i.e., students, teachers, and school staff) and that aim to reduce the population burden of mental health conditions. There is also growing emphasis on the interface between educational attainment and health. For example, FRESH aims to promote safe learning environments and improve children’s health skills with the goal of improving the quality of education delivered. UNICEF’s Child-Friendly Schools initiative focuses on the wellbeing of the whole child, including attention to the different needs of different groups according to gender, physical ability, and socio-economic status, appreciating the importance of these for health and education. The Global Standards for Health-promoting Schools encourages schools to create the type of social and learning environments that support respectful relationships between students and warm, engaging relationships between students and teachers (15, 54). The whole-school approach advocated within Health-promoting schools moves beyond a singular or narrow focus on health education to one that aligns curriculum approaches with efforts to build social and emotional literacy, positive peer-peer and student-peer relationships, and inclusive school communities with the knowledge that these are all reflected in mental health and wellbeing (55, 56). Schools are learning environments that, year by year, month by month, and even day by day, build on earlier investments that extend students’ engagement and capabilities, consistent with knowledge of their cognitive, social, and emotional capabilities. In this way, schools are inherently oriented to healthy growth and development based on incremental approaches to learning (17). Arguably, universal approaches to health promotion are highly consistent with this educational philosophy. While feasible for schools to implement, such universal approaches to mental health promotion require intentional, thoughtful, and measured approaches, just as schools require to achieve their learning objectives (16). These global documents encourage government agencies to contribute to the establishment of school health and support the sustainability of schools’ universal mental health programs by facilitating policies and regulations, funding, and resources. Overtly framing universal health initiatives around educational objectives may help schools and their staff to appreciate some of the important intersections between educational and health goals (e.g., good nutrition, physical activity, sleep).

These UN agency guidelines and manuals consistently reinforce the importance of supporting schools to provide an enabling context for mental health and wellbeing. Within a comprehensive school-health framework, the expectations are that schools invest in building their internal and external capacities around a variety of roles. These include developing intersectoral partnerships. Every school community needs access to referral networks, resources, and health professionals who are trained to diagnose and treat common mental disorders, whether those services are delivered within the school or beyond its walls (57). Embodying health and education sector expertise within school-health committees is also required. However, wider partnerships are needed for schools to become safe, non-punitive, and inclusive learning environments, including transport and welfare. Given their thought leadership in many communities, partnering with religious leaders will also be valuable for schools, as efforts to promote mental health are highly reliant on reducing the stigma around mental disorders (58, 59).

Within this global policy review, it is not surprising that the various manuals were oriented around school-led programs and focused on the delivery system using a school-led or “bottom-up approach.” In this context, schools initiate implementation because they seek specific education, social, or health benefits, which is commonly followed by efforts to seek support from community leaders and policymakers to promote sustainability. Many previous approaches to mental health promotion within a Health-promoting Schools framework started with school-level initiatives. For example, WHO’s guidance on suicide prevention (SUPRE) in schools was launched within a Health-promoting Schools approach in 1998 (60). Since then, guidelines for countries to develop national SUPRE plans and strategies have been developed as a more “top-down” approach (61, 62) consistent with the recent guidelines we reviewed that emphasize the importance of building a system for mental health promotion. These approaches recognize the importance of government investments that support schools in implementing specific actions. For example, FRESH is a top–down strategy that encourages governments to initiate and develop policies on school health, including creating a school-health system, and then working with schools to implement this approach.

Similarly, WHO’s school-based violence prevention is a practical approach that targets schools to support them to implement the earlier guideline of the Global Plan of Action on violence against women and children that had been accepted by the national governments of UN member states (35, 40, 60). Rather than individual schools taking responsibility for designing or developing mental health-promoting interventions, these top-down policies view schools as the extended arm of the government with the responsibility for implementation of the national health system or plan. While national accountability for school mental health promotion could be equally advocated through a top-down approach, the lack of a consistent approach to monitoring and accountability in these documents was disappointing.

It is hoped that, building on evidence of effectiveness, these findings can help guide national governments and schools in their decisions about what to target within school mental health promotion, which strategies they might select and what partnerships are required. However, this review also has wider learnings around the inconsistent use of language, whether around the meaning of “comprehensive school health” or indeed around the definition of a manual or guideline. We suggest that manuals should aim to support school initiatives in developing and implementing school-based mental health promotion initiatives; manuals should therefore be practically oriented and focus on troubleshooting or problem-solving. Guidelines can be less practical, as their top–down approach is intended to assist governments in making plans, facilitating policies, allocating resources, and ensuring monitoring strategies are in place and, in turn, will inform the next steps. We found that across these documents, very few manuals provided sufficient practical support to schools that would enhance their “bottom-up” role in providing enabling environments for mental health and wellbeing. This may suggest that manuals are more relevant at a country level, rather than globally. Regardless, beyond the need for both top–down and bottom–up approaches, individual guidelines and manuals should be supported by training materials and assessment tools for monitoring implementation and evaluating outcomes.

This study has several limitations. Firstly, notwithstanding active discussion between the authors of any questionable findings, the document searches and data extraction were identified by a single reviewer as part of the first author’s doctoral studies. Reliability might have been enhanced if more than one reviewer had been involved in screening and data extraction. Secondly, this global policy review did not extend beyond the network of UN agencies. Including global professional association documents may have widened the scope of identified documents. However, as these UN agency documents are evidence-based, we anticipate that they will have built on any important, relevant documents. Thirdly, we did not set out to capture country-level guidelines and manuals due to the challenge of identifying these and the variety of languages in which they are published. We fully expect that at least some of these would be relevant globally. Fourthly, the majority of documents were produced by the WHO. On the one hand, this is logical, given WHO is the primary global body managing health issues. Arguably, more documentation from UNESCO may have been expected given their school policy context. The predominance of WHO documents could also reflect the search strategy that used WHO-IRIS, a WHO library platform. Most health-related policy documents should be stored in this platform, regardless of the UN agency that produces them, but our inclusion of two additional search approaches was with the intention of mitigating this potential bias. Finally, we did not set out to evaluate the effectiveness of mental health promotion in schools, nor the costs of implementing any of these approaches, both of which are important to explore in further studies.

## Conclusion

This policy review reveals that the UN agencies have made significant efforts to orient national governments and their schools to the importance of promoting the mental health and wellbeing of school-age children worldwide. Notwithstanding inconsistent terminology between guidelines and manuals, and a variety of ways that comprehensiveness could be conceptualized, we found that the scope of these guidelines and manuals was predominantly oriented toward universal interventions using the approach of Health-promoting Schools. While the global standards for Health-promoting Schools include a set of indicators, these do not focus on specific health topics such as mental health (15). Developing a set of indicators for specific health topics, such as mental health promotion, could be helpful for national governments, as this would drive accountability through monitoring and evaluation.

From the policy perspective, this review reinforces the importance of government, international agencies, and donors developing plans that support schools to provide enabling learning and social environments that promote mental health and wellbeing, which will benefit from both top-down and bottom-up approaches. An initial priority is ensuring that country-level policymakers create policies, operational guidelines, and regulations to facilitate how schools address the spectrum of mental health promotion, including health promotion, prevention, and the provision of health services for those who need them, whether at school or in the community.

## Author contributions

MM led this research as part of her doctoral thesis at The University of Melbourne, which is supervised by SS, PA, and JF. MM contributed most to the research and writing of the manuscript. PA served as the second supervisor, outlined the concept, helped with writing ideas, and co-authored the abstract. JF served as the third supervisor, provided guidance throughout execution of the project, provided feedback on the writing draft, and co-authored the abstract. SS served as the primary supervisor, outlined the concept, secured funding and provided guidance throughout execution of the project, and co-authored the abstract, background, and method. All authors contributed to the article and approved the submitted version.

## Funding

The publication of this manuscript was funded by the Centre for Adolescent Health, Royal Children’s Hospital, Parkville, VIC, Australia. MM was funded by the Australia Awards Scholarships from the Government of Australia.

## Conflict of interest

SS contributed to the development of the WHO and UNESCO Global Standards and Systems for Health-Promoting Schools. SS was a member of the WHO Technical Advisory Committee for School Health Services.

The remaining authors declare that the research was conducted in the absence of any commercial or financial relationships that could be construed as a potential conflict of interest.

The handling editor SS declared a past collaboration with the author SS.

## Publisher’s note

All claims expressed in this article are solely those of the authors and do not necessarily represent those of their affiliated organizations, or those of the publisher, the editors and the reviewers. Any product that may be evaluated in this article, or claim that may be made by its manufacturer, is not guaranteed or endorsed by the publisher.

## Footnotes

^1^ https://librariesaustralia.nla.gov.au
