# Supplementary material for: School-based mental health promotion: A global policy review
Source: Front Psychiatry. 2023 Apr 17;14:1126767. doi: 10.3389/fpsyt.2023.1126767 (PMC10149729; doi:10.3389/fpsyt.2023.1126767)
Supplement: Supplementary file 1 [file Data_Sheet_1.PDF]

# SUPPLEMENTARY BOXES

## Box 1. Search strategy

Keywords used to explore Google Scholar, WHO-IRIS library (https://apps.who.int/iris/), and the National Library of Australia (https://librariesaustralia.nla.gov.au) for published documents produced by UN agencies (i.e. WHO, UNESCO, UNICEF, UNFPA) within the period from 2000 to 2021. The search was conducted from May 1st 2021 to May 16th 2022.

***Libraries Australia - National Bibliographic Database (ANBD)***
Title: school* AND school health OR health promoting school OR mental health OR mental illness* OR mental disorder* OR psychosocial* OR psychologi* OR wellbeing OR well-being AND manual* OR guide* OR policy* OR framework* AND WHO (any of these) AND Author: WHO (exact match) AND Subject: health OR mental health OR wellbeing OR well-being OR psychosocial OR school health OR school* OR child* OR adoles* OR student* OR manual* OR guideline* OR framework OR policy* (any of these)

Title: school* AND school health OR health promoting school OR mental health OR mental illness* OR mental disorder* OR psychosocial* OR psychologi* OR wellbeing OR well-being AND manual* OR guide* OR policy* OR framework* AND UNICEF (any of these) AND Author: UNICEF (exact match) AND Subject: health OR mental health OR wellbeing OR well-being OR psychosocial OR school health OR school* OR child* OR adoles* OR student* OR manual* OR guideline* OR framework OR policy* (any of these) NOT Any keyword: NOT country report* NOT book* NOT report* NOT brief* NOT editorial* NOT research* NOT article* NOT research article* NOT book* NOT journal* NOT speech* NOT resolution NOT technical consultation NOT statistic* report* Language: English Year range: 2000-2021 (any of these)

Title: school* AND school health OR health promoting school OR mental health OR mental illness* OR mental disorder* OR psychosocial* OR psychologi* OR wellbeing OR well-being AND manual* OR guide* OR policy* OR framework* AND UNESCO (any of these) AND Author: UNESCO (exact match) AND Subject: health OR mental health OR wellbeing OR well-being OR psychosocial OR school health OR school* OR child* OR adoles* OR student* OR manual* OR guideline* OR framework OR policy* (any of these) NOT Any keyword: NOT country report* NOT book* NOT report* NOT brief* NOT editorial* NOT research* NOT article* NOT research article* NOT book* NOT journal* NOT speech* NOT resolution NOT technical consultation NOT statistic* report* Language: English Year range: 2000-2021 (any of these)

Title: school* AND school health OR health promoting school OR mental health OR mental illness* OR mental disorder* OR psychosocial* OR psychologi* OR wellbeing OR well-being AND manual* OR guide* OR policy* OR framework* AND UNFPA (any of these) AND Author: UNFPA (exact match) AND Subject: health OR mental health OR wellbeing OR well-being OR psychosocial OR school health OR school* OR child* OR adoles* OR student* OR manual* OR guideline* OR framework OR policy* (any of these)

***WHO IRIS library***
school* OR education* OR health OR mental OR school health OR health promoting school* OR mental health OR mental illness* OR mental disorder* OR psychosocial* OR psychologi* OR wellbeing OR well-being OR (manual* OR guide* OR policy* OR framework* NOT country report* NOT report* NOT brief* NOT editorial* NOT research* NOT article* NOT speech* NOT resolution NOT technical consultation) AND (child* OR adolescen* OR student* OR school-age*) Author:(WHO OR world health organization NOT country report* NOT regional report*) Language: English NOT Spanish NOT French NOT Russian NOT Arabic NOT Vietnamese NOT Japanese
filter Language: English

***Google Scholar***
"mental health" AND wellbeing AND well-being AND psychosocial AND "school health" AND school* AND child* AND adoles* AND student* AND manual* AND guideline* AND policy* AND framework* author:"world health organization" source:"world health organization" OR WHO language: English Year range: 2000-2021

"mental health" AND wellbeing AND well-being AND psychosocial AND "school health" AND school* AND child* AND adoles* AND student* AND manual* AND guideline* AND policy* AND framework* author: ”united nations international children's emergency fund” OR UNICEF source: :”united nations international children's emergency fund” OR UNICEF language: English Year range: 2000-2021

"mental health" AND wellbeing AND well-being AND psychosocial AND "school health" AND school* AND child* AND adoles* AND student* AND manual* AND guideline* AND policy* author:"united nations educational scientific and cultural organizations" OR UNESCO source:"united nations educational scientific and cultural organizations" OR UNESCO language: English Year range: 2000-2021

"mental health" AND wellbeing AND well-being AND psychosocial AND "school health" AND school* AND child* AND adoles* AND student* AND manual* AND guideline* AND policy* NOT research article author:"united nations population fund" OR UNFPA source: UNFPA language: English Year range: 2000-2021

**Box 2. Global school mental health manuals and guidelines identified from the UN agencies, 2000-2021 (n=16)**

1. WHO (2000). Preventing suicide: A resource for teachers and school staff.
2. WHO (2000). Local action: Creating health promoting schools.
3. UNESCO (2002). FRESH: A comprehensive school health approach to achieve Education For All.
4. WHO (2003). Creating an environment for emotional and social wellbeing. An important responsibility of a health-promoting and child friendly school.
5. WHO (2003). Family Life, reproductive health and population education: Key elements of a Health Promoting Schools.
6. WHO (2003). Skills for health: Skills-based health education, including life skills: An important component of a child-friendly/health-promoting school.
7. UNICEF (2009). Child friendly schools manual.
8. UNESCO (2016) Education for health and wellbeing: Contributing to the Sustainable Development Goals.
9. WHO (2016). INSPIRE: Seven strategies for ending violence against children.
10. WHO (2016). Mental Health Gap Action Programme (mhGAP) Intervention Guide for mental, neurological and substance use disorders in non-specialized health settings. Version 2.0.
11. UNICEF (2018). Operational guidelines on community-based mental health and psychosocial support in humanitarian settings: Three-tiered support for children and families.
12. WHO (2019). Accelerated Action for the Health of Adolescents (AA-HA!) A manual to facilitate the process of developing national adolescent health strategies and plans.
13. WHO (2019). School-based violence prevention: A practical handbook.
14. WHO (2020) Helping Adolescents Thrive (HAT): Guidelines on mental health promotive and preventive interventions for adolescents.
15. WHO (2021). Making every school a health-promoting school: An implementation guidance.
16. WHO (2021) Guideline on School Health Services.
